# Symptomatic Carotid Free-Floating Thrombus: About Management of 50 Cases in a Referral Neurovascular Center

**DOI:** 10.3390/jcm12237238

**Published:** 2023-11-22

**Authors:** Sarah El Harake, Emilie Doche, Julien Bertolino, Nadia Laksiri, Michel Alain Bartoli, Barbara Leclercq, Laurent Suissa, Gabrielle Sarlon-Bartoli

**Affiliations:** 1 Vascular Medicine Department, APHM, Timone Hospital, 13005 Marseille, France; sarah.elharake@ap-hm.fr (S.E.H.); julien.bertolino@ap-hm.fr (J.B.); barbara.leclercq@ap-hm.fr (B.L.); 2 C2VN, Faculty of Medecine, Aix-Marseille University, 13284 Marseille, France; 3 Neurovascular Department, Stroke Center, APHM, Timone Hospital, 13005 Marseille, France; emilie.doche@ap-hm.fr (E.D.); nadia.laksiri@ap-hm.fr (N.L.); laurent.suissa@ap-hm.fr (L.S.); 4 Vascular Surgery Department, APHM, Timone Hospital, 13005 Marseille, France; michel.bartoli@ap-hm.fr

**Keywords:** free-floating thrombus, carotid, atheroma, hypercoagulability

## Abstract

Background: Carotid free-floating thrombus (CFFT) is an uncommon disorder. The aim of this study was to describe a French cohort of CFFT patients. Methods: We conducted a retrospective monocentric study from a Stroke Center among patients admitted for stroke with CFFT. Results: Between January 2017 to December 2019, 2038 ischemic strokes were recorded. A total of 50 patients with CFFT were consecutively included (32 men/18 women). The mean age was 58.2 years (±11.7). Their etiologies were atheroma (46%), carotid dissection and web (20%), hypercoagulability disorders (16%) and arrhythmia (10%). Exclusive medical management was performed in 38 patients (76%): 29 (59.2%) were anticoagulated and 9 (18.4%) received antiplatelets alone in the first week. Surgical intervention was performed in the first 30 days for 11 patients (22%). The main surgical indication was a residual carotid stenosis over 70%. Only three patients had a recurrent stroke in the medical group with anticoagulants. No patients in the antiplatelet group or the surgical group had a recurrent stroke. Conclusions: Our study summarized a large cohort of 50 patients with CFFT. This diagnosis implies the need to search for a local arterial disease and to screen for hypercoagulability states. An initial medical strategy followed by a delayed carotid surgery if the follow-up imaging shows a residual stenosis appears to be safe.

## 1. Introduction

Carotid free-floating thrombus (CFFT) is a rare and understudied situation. Its definition is debated, but the literature suggested that it is an elongated thrombus partially attached to the carotid arterial wall which can move according to the blood flow and without complete intraluminal occlusion [1]. Because of a lack of a unified definition, the literature is poor. However, computed tomography angiography improves the diagnosis with the “donut sign” in the cross section [2]. It is reported in 1.3% of ischemic stroke patients [3]. The most common etiology is atherosclerotic, but CFFT was also related to the prothrombotic state, dissection, fibromuscular dysplasia and vasculitis [1]. The relative proportion of atherosclerotic versus non-atherosclerotic causes of FFT is unknown. Regarding the natural history of CFFT, CFFT is a high risk for stroke recurrence and potential outcomes include progression to occlusion, distal embolization, stabilization or disappearance. CFFT was associated with a recurrent risk of stroke and the optimal management is still unclear, with no RCTs to guide practice. In a meta-analysis of 58 case series and 83 case reports [3], 345 patients were treated with “antithrombotic” or “interventional” methods, in whom 30-day death, TIA/stroke or silent ischaemia on MRI was 17.1% (95% CI 13.1–21.1), with a 30-day risk of stroke/death of 11.1% (95% CI 7.7–14.3) *p* = 0.54. These high event rates presumably reflect high rates of cerebral embolization. In a Cox regression analysis of relatively poor data, neither anticoagulation versus no anticoagulation (HR 1.21; 95% CI 0.35–4.23, *p* = 0.76) nor interventions < 3 days versus >3 days after symptom onset (HR 0.78; 95% CI 0.24–2.57), *p* = 0.69) were associated with different risks of silent ischemia, TIA or stroke/death at 30 days [3]. However, patients with FFT undergoing thrombolysis had higher rates of silent ischemia, TIA or stroke/death (HR 14.79; 95% CI 3.41–64.25) *p* < 0.001) [3]. The 2021 SVS, AHA and ESO guidelines provide no advice about the management of symptomatic patients with FFT. The 2021 German–Austrian guidelines advise that (in selected patients) CEA or CAS should be performed within the first hours of the index event after consultation with stroke specialists [4]. Finally, the recently published Guidelines by ESVS 2023 tried to give a possible management strategy and strongly recommend anticoagulation (Class I, Level C) [5].

The aim of this study was to describe the presentation, the diagnostic modalities and the management of a French cohort of CFFT from a stroke referral center. Our objective was to highlight the different etiologies and the best therapeutic strategy to avoid ischemic recurrence.

## 2. Materials and Methods

### 2.1. Study Design

We conducted a retrospective monocentric study between January 2017 and December 2019. We collected the following clinical data on patient’s medical records from the Neurovascular Units (NVUs) of our center, The Timone Hospital in Marseille.

### 2.2. Patient Selection

We included patients presenting with stroke or transient ischemic attack and the presence of CFFT on the computed tomography defined by a filling defect in two axial planes: the “donut sign” and “the finger sign” [3] and confirmed by a multi-disciplinary medical expert. We excluded hemorrhagic strokes, ischemic strokes with initial hemorrhagic conversion, carotid occlusion, free-floating thrombus of other cerebral arteries and cerebral tumors.

### 2.3. Data Collection

A database was created to record the following data: age, sex, cardiovascular risk factors including diabetes, hypertension, dyslipidaemia (treated dyslipidaemia or ldlc > 1.4 g/L), cardiovascular heredity (myocardial infarction in a first degree relative of male sex occurring before the age of 55 years or of female sex occurring before 65 years), smoking and toxic consumption, past medical history and history of cardiovascular events, including stroke, coronary heart disease and peripheral vascular disease. Current patients’ treatments were also recorded, including the type of antiplatelet or anticoagulants.

For diagnostic and aetiologic research, all patients had cerebral imaging (Magnetic Resonance Imaging (MRI) or Computed Tomography (CT scan)) and a carotid artery imaging characteristic ultrasound carotid was performed when it was available.

Laboratory blood tests were performed with blood count, C-reactive protein, blood ionogram, hemostasis tests and low-density lipoprotein (LDL) cholesterol. For patients under 50 years old or without underlying pathology, the laboratory findings included thrombophilia search with antiphospholipid antibodies (aPLs), and inherited thrombophilia, homocysteine and b9–b12 vitamins and JAK2 V617F mutation.

Cardio embolic explorations were also performed for all the patients: ECG, echocardiography, transesophageal echocardiography and 24 h Holter monitoring.

For some patients, a thoraco-abdominal CT scan was performed to look for neoplasia when suspected.

We classified management based on whether a patient only received antiplatelet therapy, or anticoagulant, during the first week or underwent surgery during the first 30 days (surgical group). Medical treatment was recorded in two sub-groups: anticoagulation and antiplatelet therapy. Anticoagulation therapy was heparin, warfarin, or oral anticoagulant. Antiplatelet therapy was aspirin or clopidogrel. Patients receiving both anticoagulation and antiplatelet therapy were considered to be part of the anticoagulation group.

#### Follow Up

We gathered the CT-scan follow-up on the 7th day and between 3 and 6 months. They were classified as complete dissolution and persistent or recurrent CFFT (same aspect on imaging or recurrence after disappearing). Length of follow up and clinical outcomes (mortality, ITA/stroke recurrence, or “loss of follow up”) were also collected. Patients’ clinical outcomes were recorded: recurrence of stroke or TIA, neurological recurrence (including worsening of neurological symptoms or occurrence of epilepsy), cerebral bleeding, or death. We compared clinical and imaging characteristics and outcome in the two groups: medical versus surgical.

### 2.4. Ethical Approval

This study received authorization from the National Data Protection Commission (CNIL) and has been declared to Access Portal for Health Data of the Public Assistance of Marseille Hospitals.

### 2.5. Statistical Analysis

Descriptive and comparative statistics were performed with Prism software (Version 5.01, Prism Software Corp, Irvine, CA, USA). A value of *p* < 0.05 was considered as significant. Quantitative variables are described using mean (±standard deviation (SD)) and qualitative variables as numbers (%). Univariate comparisons between groups used Student’s *t* tests or Mann–Whitney tests, as appropriate, for quantitative variables and Pearson’s χ^2^ tests or Fisher’s exact tests, as appropriate, for qualitative variables.

## 3. Results

### 3.1. Characteristics of Patients

Between January 2017 and December 2019, 2038 ischemic cerebral strokes were recorded. All patients presented with neurological symptoms. A total of 50 patients with CFFT met our inclusion criteria (incidence 2.45%). Demographic data are described in Table 1.

The studied group included 32 men (64%) and 18 women (36%), with a sex ratio of 1.77. The mean age was 58.2 years (±11.7). Cerebral zone infarctions were visualized on 48 cerebral MRI and on two CT imaging scans. There were 47 unilateral infarct lesions (94%) and three bilateral lesions (6%). Regarding the cardiovascular risk factors, we noted a tobacco use in 27 patients (54%). A total of 20 patients (40%) had hypertension, 20 (40%) had dyslipidemia and 9 (18%) had diabetes. Four patients (8%) declared cannabis use.

Assessing the current medical treatment, eight patients (16%) were treated with antiplatelet therapy and six (12%) with anticoagulants. No patient was treated by both anticoagulant and antiplatelet therapy.

On CT angiograms, the localization of CFFT was the cervical internal carotid artery for 31 patients (62%), the carotid bulb for 11 (22%), the intracranial part for 7 (14%) and the common carotid for 1 (2%).

### 3.2. Associated Pathologies

Underlying pathologies were atheroma in 23 cases (46%) and carotid dissection and web in 10 cases (20%) (Table 2).

Patients with atheroma had a mean age of 59.3 years (±10.8), mainly male (78%) with cardiovascular risk factors: hypertension (12/23; 52%), dyslipidemia (10/23; 43%), tobacco use (10/23; 43%) and diabetes (8/23; 35%).

Patients with dissection or web had a similar mean age of 58.2 years (±11.7), but there were mostly women (60%) with fewer cardiovascular risk factors: hypertension (2/10; 20%), dyslipidemia (4/10; 40%), tobacco use (2/10; 20%) and diabetes (0/10; 0%).

Thrombophilia testing was performed for 32 patients (64%) and was abnormal for 7 patients (22%). Hypercoagulability disorder was noted for eight patients (16%): myeloproliferative neoplasia in six patients, antiphospholipid syndrome for one and bladder cancer for another. Arrhythmia represented five patients in our cohort (10%). Cannabis consumption was incriminated in one case. For one patient, the cause of CFFT was unknown and for two patients, the diagnosis was uncertain: one with a myelodysplastic disorder and another with monoclonal B cells lymphocytosis.

### 3.3. Management and Outcomes

For this section we analyzed 49 patients, because one 72-year-old woman died a few hours after her admission (acute respiratory distress) and was not analyzed. Data are reported in Table 3.

Exclusive medical management was performed in 38 patients (76%). Out of 38 patients medically treated, 29 (59.2%) were anticoagulated with or without antiplatelet therapy and 9 (18.4%) received antiplatelet alone in the first week.

At 7 days of initial treatment, we noted a complete regression of the CFFT for 4 patients (44.4%) of the antiplatelet group and 17 (58.6%) of the anticoagulation group.

Surgical intervention was performed in the first 30 days for 11 patients (22%). The surgical indication was a residual carotid stenosis of > 70% for eight patients (72.7%) and a persistent CFFT for three (27.3%). In the first week of management, out of the 11 patients, 10 were treated with anticoagulants (90.9%), including 7 with an associated antiplatelet agent. Only one patient was treated with an antiplatelet agent alone.

Regarding clinical outcomes, the mean follow-up was 8.9 months (±6.6). Only three patients had a recurrent stroke in the medical group with anticoagulants. These patients had a dissection, a web and atheroma. No patients in the antiplatelet group and surgical group had a recurrent stroke. One patient in the antiplatelet group presented a neurological recurrence, along with two patients in the anticoagulant group. These differences were not significant.

The 3–6 months radiological follow up noticed a recurrent CFFT in two cases regardless of etiology, surgical/medical groups or antiplatelet/anticoagulant drugs (*p* = NS).

## 4. Discussion

The CFFT is a rare condition described for the first time in 1905 in a report by Chiari, where he describes, on postmortem, an elongated 1.5 cm intraluminal thrombus as the probable source of a patient’s stroke [1]. Thereafter, less than 600 case reports were recorded in the literature. The incidence is uncertain and depends on how CFFT was defined on imaging (angiography, CT-scan or Duplex Ultrasound (DU)). One retrospective study with cerebral angiographies reported an incidence of 1.45% (29 carotid free-floating thrombi on 2000 angiographies from 1975 to 1985) [6]. Another study with DU imaging of the carotid arteries demonstrated an incidence of 0.05% [7]. A more recent review of the literature reported 1.3% of CFFT in stroke patients [3]. Our study analyzed a French monocentric cohort of patients with CFFT diagnosed using CTA: the incidence was 2.45%, highlighting 50 CFFT in 2038 strokes. It is the highest incidence to date, after the Buchan et al. publication (1.45%) [6]. As Fridman et al. pointed out, the patient-weighted frequency of CFFT in stroke patients was 0.4% before the year 2000 and 1.6% after 2000 [3]. Indeed, historically, CFFT were diagnosed using angiography, but the development of non-invasive and high-resolution vascular imaging with CTA had permitted the increase in the diagnosis of CFFT over time. A retrospective review with DU imaging of the carotid arteries demonstrated an incidence of 0.05% (1/2000) [7]. In addition to imaging studies, FFT may be misidentified intraoperatively as an atherosclerotic plaque instead of a thrombus, thereby contributing to the underestimation of the incidence of this entity.

Angiography was used heavily in the diagnostic phase of FFT. This may reflect the historic bias of obtaining angiograms for the carotid disease, as many of the papers predate the era of DU imaging. When more than one modality was used, findings were congruent, suggesting angiography is not mandatory and that CTA, MRA, or DU imaging are sufficient. Despite one case report where a stroke was precipitated during a DU examination of FFT, DU imaging remains the safest and least invasive study [8].

The localization of CFFT was few described in the literature. In our study, the most common localization was the cervical internal carotid artery (62%). However, 14% of CFFT was localized in the intracranial part of the internal carotid. This may explain why ultrasound is less efficient than CTA for the detection of these thrombi because the intracranial region cannot be explored precisely.

In our cohort and similarly to the literature [3], the epidemiology of CFFT concerned young patients with a mean age of 58 years and more frequently male. The higher preponderance in males can be explained because of the prevalence of carotid atherosclerosis in the general population [9]. Almost all of the patients reported in the literature were symptomatic. The natural history of either symptomatic or asymptomatic patients is not known, although one would expect it to be adverse in the former. The most common underlying situation of our cohort was atheroma with 46%. These patients were mainly men with a mean age of 59 years and cardiovascular risk factors. The second was dissection and web, in mostly women with a mean age of 58 years and fewer cardiovascular risk factors. Hypertension and smoking are dissection risk factors. However, fibromuscular dysplasia is also a cause of dissection or web and should be investigated, especially in women without cardiovascular risk factors [10].

Hypercoagulability states overlap hematological disorders, acquired or congenital thrombophilia and cancer. These prothrombotic states are reported to be more frequent in strokes caused by CFFT than strokes without CFFT (18% against 4%, *p* < 0.01) [11]. The hypercoagulability state was also associated with CFFT in the literature (47%) [1]. In our study, we noted only 16% of hypercoagulability disorders. But it is interesting to note that out of 32 patients tested, we diagnosed a thrombophilia for 7 (6 with myeloproliferative neoplasia and 1 with an antiphospholipid syndrome). These findings support the importance of testing for thrombophilia in cases of CFFT. Cardioembolism and toxic consumption should be researched too. Since the beginning of the COVID-19 pandemic, several cases of CFFT during COVID-19 infection were described [12,13]. In a recent publication, up to 20% of carotid FFT had a non-atherosclerotic etiology [14]. Some studies voluntarily excluded arterial pathologies to highlight these minor causes [11,15].

About the management, there is no consensus. Medical and surgical strategies have both been used, with neither clearly superior to the other [1]. This was underlined in our study: no significant difference between clinical and radiological outcome in any of these strategies was demonstrated. However, a CFFT management’s evolution can be advantageous over the years. The dictum of immediately operating on symptomatic CFFT, originally described, may need to be re-evaluated. In fact, the complete dissolution of the CFFT without any further neurologic progression occurred in more than 80% of patients treated medically [1]. In our study, 4% of patients had a recurrent stroke against 28% in Buchan’s population [6]. A total of 90% of our patients did not present recurrent stroke during the follow up, against 72% in Buchan’s population [6]. Regarding the surgical group, no patient in our study had a recurrent stroke, whereas 37.5% of Buchan’s 16 patients suffered from a recurrent stroke. The main surgical strategy difference was the time of the surgery which was performed during the early stage of the disease (average at 74.19 ± 9.68 h (range 2–312 h) in Buchan‘s study against 282 ± 132.84 h (range 6–456 h) in our experience [6]. The main operative indication in our surgical population was a significant carotid stenosis over 70% according to the NASCET classification, rather than the CFFT presence. The early onset of surgery was probably responsible for the “over-morbidity” observed in Buchan’s population (37.5% of TIA/stroke). Thus, we could suggest that an initial medical strategy followed by a delayed carotid surgery if the follow up imaging shows a residual stenosis or a persistent CFFT appears to be a safe and optimal management of the CFFT. In our study, we did not find any difference for early anticoagulation against an antiplatelet therapy alone in reducing the short-term risk of a recurrent stroke or death. The 2021 SVS, AHA and ESO guidelines provide no advice about the management of symptomatic patients with FFT. The 2021 German–Austrian guidelines advise that (in selected patients) CEA or CAS should be performed within the first hours of the index event after consultation with stroke specialists [4]. Finally, the recently published Guidelines by ESVS 2023 tried to give a possible management and strongly recommend anticoagulation (Class I, Level C) [5].

In the Fridman et al. study, an increased risk of ischemic stroke recurrence in patients who received IV rt-PA was reported [3]. In our study, of the nine patients who received IV rt-PA, one patient died early and one presented a stroke recurrence. There was no significant difference with the group without thrombolysis, but there was a tendency for a worse prognosis (*p* = 0.1).

Our study limits were the same as for all retrospective monocentric studies with a single investigator. The higher incidence of the CFFT in our study can be explained by the young average age of admitted patients in our neurological intensive care unit and the CTA scan earliness of realization.

## 5. Conclusions

Our study summarized the monocentric management of 50 patients with CFFT. Similar to the literature, these patients are young and predominantly male, with atheroma carotid plaque. Almost all of the patients are symptomatic. All initial diagnostic modalities were congruent and therefore probably equivalent, in the delineation of CFFT. This may obviate the need for confirmatory studies such as cerebral angiography unless otherwise warranted. The CFFT diagnosis implies the need to search for a local arterial disease in the first step (atheroma, dysplasia, dissection, web). Screening hypercoagulability states including hematologic malignancies, thrombophilia and cancer is also important. Regarding the management, despite the lack of a consensus, the dictum of immediately operating a symptomatic CFFT may need to be re-evaluated. Currently, the safest management is medical with a lower recurrence rate. Carotid surgery is secure and indicated after medical therapy, if residual carotid stenosis is over 70% NASCET or for persistent CFFT.

## Figures and Tables

**Table 1:** Demographic, clinical and biological data of the studied population of patients.

	Population *n* = 50 (%)
Epidemiological characteristics	
Age (years)	58.2 (±11.7)
Male sex/Female sex	32 (64%)/18 (36%)
Cardiovascular risk factors	
Smoker	27 (54%)
Hypertension	20 (40%)
Dyslipidemia	20 (40%)
Diabetes	9 (18%)
Previous stroke	9 (18%)
Coronary Heart Disease	4 (8%)
Cannabis use	4 (8%)
Usual treatments	
Antiplatelet	8 (16%)
Anticoagulant	6 (12%)
Antihypertensive therapy	16 (32%)
Cholesterol-lowering drugs	13 (26%)
Localization of CFFT	
Common carotid artery	1 (2%)
Carotid bulb	11 (22%)
Cervical internal carotid artery	31 (62%)
Intracranial part carotid artery	7 (14%)

Quantitative variables are described using mean (±SD); CFFT: carotid free-floating thrombus.

**Table 2:** Underlying pathologies.

	Population *n* = 50 (%)
Arterial disease	33 (66%)
Atheroma	23 (46%)
Dissection	8 (16%)
Web	2 (4%)
Hypercoagulability disorder	8 (16%)
Myeloproliferative neoplasia JAK 2	5 (10%)
Thrombocythemia JAK 2 negative	1 (2%)
Antiphospholipid syndrome	1 (2%)
Solid cancer (bladder cancer)	1 (2%)
Cardioembolic disease	5 (10%)
Atrial fibrillation	5 (10%)
Idiopathic or uncertain cause	4 (8%)
Cannabis use	1 (2%)
Monoclonal B cells lymphocytosis	1 (2%)
Myelodysplastic syndrome	1 (2%)
Idiopathic	1 (2%)

**Table 3:** Comparison of medical groups and surgical group.

	Medical Group Antiplatelet *n* = 9 (18.4%)	Medical Group Anticoagulation *n* = 29 (59.2%)	Surgical Group *n* = 11 (22.4%)	*p*
Age	60.1 (±11.1)	58.2 (±11.7)	59 (±10.5)	NS
Sex (Male/Female)	7 (78%)/2 (22%)	17 (58.6%)/12 (41.4%)	8 (72.7%)/3 (27.3%)	NS
Previous stroke	3 (33.3%)	4 (13.8%)	2 (18.2%)	NS
Initial treatment of the stroke *	5 (55.6%)	7 (24.1%)	6 (54.5%)	NS
Thrombolysis	2 (22.2%)	4 (13.8%)	5 (45.5%)	
Thrombectomy	4 (44.4%)	4 (13.8%)	5 (45.5%)	NS
Thrombus evolution at J7				
Complete Thrombus regression	4 (44.4%)	17 (58.6%)	0	0.0037
Stenosis > 60%	1 (11.1%)	1 (3.4%)	8 (72.7%)	<0.0001
Persistent thrombus	0	3 (10.3%)	3 (27.3%)	NS
Carotid occlusion	3 (33.3%)	8 (27.6%)	0	NS
Downstream occlusion	2 (22.2%)	2 (6.9%)	0	NS
Delay of surgery (days)	NA	NA	11.8 (±5.4)	
Treatment upon discharge from hospital				
Anticoagulation	0	14	3	
Single antiplatelet agent	9	6	1	
Double antiplatelet agent	0	2	0	
combined anticoagulant and antiplatelet	0	7	7	
Length of follow up (months)	21.6 (±12.9)	16.6 (±8.6)	16.6 (±12.2)	NS
Clinical Outcomes	*n* = 8	*n* = 18	*n* = 6	
Neurological recurrence **	1 (12.5%)	2 (11.1%)	0	NS NS
Stroke/TIA recurrence	0	3 (16.7%)	0	
Cerebral bleeding	0	0	0	
Death	0	0	0	
Loss of follow up	1	11	5	

Quantitative variables are described using mean (±SD); * some patients had both thrombectomy and thrombolysis; ** includes worsening of neurological symptoms ± occurrence of epilepsy. NS = non significant, NA = non available.

## Data Availability

Data are contained within the article.
